# Selections

**Published:** 1892-08

**Authors:** 


### *SELECTIONS.*

---

THE RESULTS OF OPERATIONS ON THE GALL-BLADDER.—Terrier (*Le Progrès Médical*) has performed eight cholecystectomies, one cholecystotomy, three cholecystostomies, one cholecystenterostomy, and one choledochotomy.

Of the eight cases of cholecystectomy seven recovered. The first was performed in 1886, and has been well since that time. In the case of cholecystotomy, the adhesions were so tight that the gall-bladder could not be sutured to the abdominal walls. This patient died. All three cases of cholecystostomy recovered.

In the case of cholecystenterostomy operation was required on account of an obstructing tumor at the head of the pancreas. The patient lived six months after surgical intervention.

Terrillon has practiced cholecystotomy with complete success in six cases, a permanent fistula in the seventh, and a failure, due to the fact that on account of adhesions it was impossible to complete the operation.

Leonté, as a result of his study of the subject, announces that surgical intervention is indicated in cases of permanent occlusion of the biliary passages; that the operation is not a serious one; that, when possible, primary cholecystectomy should be avoided, cholecystotomy being preferable; that cholecystenterostomy is indicated in cases when extirpation is not possible, and in all instances where operation is indicated the gall-bladder should be opened, since external examination through an abdominal opening is not sufficient.

Michaux performed cholecystectomy in two cases. Both resulted in cure.

Richelet performed cholecystenterostomy in one case. The gall-bladder was secured to the duodenum. The results were not very satisfactory, biliary retention persisted, and a second laparotomy was performed; the gall-bladder was opened, and an effort was made to extract the calulus. This resulted in a hemorrhage so severe that death resulted in an hour. At the autopsy it was found that the forceps had seized a calculus, which was so firmly adherent that its displacement had caused tearing of the gall-bladder, and had resulted in hemorrhage.

Boeckel reports a case of cholecystotomy operated on ten years ago and still perfectly well. The second case has remained well for two years. The third case perished. Three unsuccessful cases of cholecystostomy are also reported by this operator.—*Therap. Gazette.*

---

CONCUSSION OF THE SPINE.—Dr. P. H. Millard, of St. Paul, Professor of Surgery in the University of Minnesota, in a recent article in the *N. W. Lancet* concerning spinal injuries says if your patient is really suffering from a spinal lesion, you may expect to find one or more of the following objective symptoms to exist, to-wit:

1. Emaciation. Generally most noticeable in groups of muscle deprived of normal nerve stimuli.

2. Fibrillary twitchings and tremors of individual muscular bundles.

3. Flushings, generally confined to face or upper portion of body.

4. Heart and pulse conditions, if abnormal and not existing before injury, may reasonably be referred to the injury.

5. Ephidrosis, indicating a depressed nervous condition.

6. Cold extremities, a symptom not possible to feign.

7. Cyanosis.

8. Pupillary dilatation, indicating nervous irritation.

9. Condition of reflexes.

In conducting the examination in medico-legal cases avoid leading questions.

Let patients describe their symptoms or tell their story without the least suggestion. It is your duty to ascertain if your patient has read any work upon spinal injury since the accident, and before arriving at a definite diagnosis consider the patient's former character, especially as to veracity. It is well to make repeated examinations, as different examinations may reveal widely different results. In conclusion, you will find it impossible to arrive at your diagnosis from any one or any two symptoms, but rather from the aggregate phenomena that go to make up the clinical picture.—*Ex.*

---

WOMAN'S DISEASES.—Dr. Gertrude Harper, for thirty years a practitioner of medicine in New York, says (*Medical Examiner*): Woman's child-bearing period should not be a detriment; child-bearing is a natural function, and in my experience it has not proven to be of any more risk than men run in their daily avocations, in fact, not near as much as those (without numbers) who use tobacco and alcoholic stimulants. I have assisted more than 2,000 babies to make their début on this world's stage, yet I have *never* lost a woman in childbirth or from the effects of it. No one can point out a family where I lost a parturient woman; I have also *never* used forceps—what need of a stronger argument against the use of them than my success? I have had my share of desperate cases and narrow escapes.

The above is conclusive proof to my mind that women who die during or soon after childbirth, do so through meddlesome midwifery and mismanagement. A case in point occurred at

Haverstraw, N. Y., in 1876. I delivered a woman of a babe weighing twenty pounds, lacking four ounces ; in three weeks after her delivery she took a drive in her carriage, and she has done a phenomenal amount of walking to this date. I know of physicians who offer and advise the use of instruments ostensibly to relieve the suffering, but in reality to save time. Of course, the majority of women give their ready consent (who can blame them for wishing to be relieved), but the consequences, alas ! are usually deplorable, to put it miidly, as I have become convinced from the many cases that have applied at my office, where I am accustomed to hear a patient commence her story in the following manner : "Since my last child was born I have been thoroughly miserable. The doctor had to apply instruments," etc., etc. I am wearied with listening to these continued tales of woe, of which the use of forceps is the foundation.

It is surprising to what extent, with patience and a certain amount of reassurance, one may influence a woman in travail. I insist that no physician has a right, for his or her own selfish ends and convenience, to hasten the termination of a case by taking advantage of a woman because she implores to have her sufferings ended.

---

THE EXISTENCE OF A TONSIL SHOULD BE REGARDED AS A DISEASE.—Dr. F. H. Bosworth said before the New York Academy of Medicine, that ten or eleven years ago he had made the assertion that practically there is no tonsil in the healthy throat. The existence of a tonsil should be regarded as a disease to be dealt with summarily and promptly.

What tonsils should be cut out ? All tonsils should be cut out. Tonsils meant diseased lymphatic tissue, which if it remained a sufficient length of time would then continue to exist during life. It had been said that tonsils disappeared at puberty, but he could say they did nothing of the sort. They shrank up at puberty, but in doing so they become a source of no inconsiderable trouble, especially in forming places for the accumulation of mucus which would undergo cheesy degeneration. Continuing, Dr.

Bosworth asked, What constitutes a healthy tonsil? How were we to tell? How many children were in a state of absolute health regarding their lymphatic glands? When the glands were enlarged he assumed that they were diseased. Yet he would not say remove every tonsil. But as soon as a so-called enlarged tonsil caused symptoms he would remove it.

As to mode of operating, he condemned the electro-puncture, which sometimes required even fifty or sixty sittings, whereas a second was sufficient time by the knife. As to the knife proper, it required a wonderfully skilled surgeon to use it safely in a struggling child. He preferred the French tonsillotome to Mackenzie's. He did not consider tonsillotomy a dangerous operation; he knew of no recorded death from it by a surgeon. No surgeon would cut the carotid artery. He had seen no serious hemorrhage in the child, but in adults there was more or less perplexing bleeding, and he there employed the cold snare. As to removal of part of the tonsil only, he regarded it about as justifiable as taking off only a part of a uterine fibroid. Something was left which could be of no further use than to act as a trap for disease.—*Weekly Medical Review.*

---

MIDWIFERY AMONG THE ALASKAN INDIANS.—Dr. J. K. Simpson, of Alaska, gives, in a recent number of the *Occidental Med-Times*, a sketch of the obstetric customs of the Alaskan Indians. His observations were made in the southeast of Alaska. When a woman arrives at full term a tent or hut is erected, and a hole dug in the middle and lined with moss. When labor commences the woman goes to the hut and squats over the hole, as in the act of defecation, grasping a pole driven into the ground in front. She is attended by three squaws; one sits behind her, and when a pain comes on clasps her arms firmly about the abdomen, while the other two women press firmly with their shoulders against the knees of the parturient woman. The child drops into the hole, occasionally breaking a bone or sustaining other injury. The umbilical cord is divided about four inches from the naval by twisting it and pinching with the nails, and is not tied. The squaws maintain their relative positions during the third stage of labor; a binder, consisting of two pieces of cloth or skin quilted together, and strengthened by pieces of bark, is applied, and the woman, if a primipara, remains where she is for ten days, but if a multipara, often goes about her work the first or second day ; in neither case is she washed for ten days, so that antiseptic midwifery is not followed. In spite of this, puerperal fever appears to be uncommon. The child, after remaining in the hole five or ten minntes, is drawn out, and the midwife dresses the stump of the cord with a foul-smelling mass consisting of the leaves of some herb chewed months before. The child's face is wiped, and it is put unwashed into a bag, stiffened with bark, which covers all but the head. Certain superstitions exist as to the placenta and cord. As a rule the placenta is burnt and the ashes carefully preserved ; when the individual dies the ashes of the placenta are placed with those resulting from the cremation of his body in a small burial house. When the stump of the cord becomes detached from the infant's navel it is enclosed in an embroidered buckskin cover and stitched to the front of the child's clothing, where it remains like a rosette until he is three or four years old. At that age the child goes into the woods and hides it.—*British Medical Journal.*

---

BACTERIOLOGY.—Bacteriology is now a science, and is very justly receiving recognition in every well equipped college and hospital. We assume that this is a fact which we need not elaborate. Hence we are surprised at the following utterance of so distinguished an authority as Dr. Theophilus Parvin, in the *Bulletin of the American Academy of Medicine* :

"The subject of bacteriology has, I believe, undue importance in professional study and teaching. Professors or demonstrators of this department of knowledge are found in many of our medical colleges, and indeed it is proposed that hospital nurses should be taught the subject—made omniscient of bacilli and cocci, and fluent in describing cultures and experimental demonstrations. Is it not possible that we may be found tithing mint and annis and cummin, and neglecting the weightier matters of law ?"

THE USE OF DIGITALIS IN AORTIC DISEASES.—Commenting upon a recent paper in the *British Medical Journal* (Mar. 12th) by Barr, Taylor argues against some of the points which Barr advanced. The latter's dictum was that digitalis is equally good in aortic and mitral disease. Taylor dissents from this view, claiming that the mechanical conditions are entirely different in the two valves. He says: There is little or no comparison between the action of the aortic valve and that of the mitral. The strain on the former is, during diastole, much heavier, and more sudden and accentuated than on the latter during systole. In other words, the closure of the aortic valve is akin to the slamming of a door, the strain commencing from the very moment at which systole ceases, whilst the opposition of the mitral folds is a gradual process, tension on the curtains to any marked extent occurring only at the end of ventricular systole, and is momentary only. And this seems to be more than emphasized by the existence in some of the lower animals not only of one tier but of several tiers of cusps in the aorta to withstand the rebound on the blood column. The avoirdupois of about the equivalent to six inches of mercury has to be considered in the aortic valve, and this is supported not only in the cusps themselves, but, as Savory has shown (and, notwithstanding the tendency of modern physiology to disturb previously accepted doctrines, this observation still seems to remain true), by the ventricle muscle itself. There is no such mechanism in the mitral valve, and therefore the incompetence of the two valves rests, to my way of thinking, on totally different physical bases. In the one it is always serious and alarming, in the other it may be, and frequently is, attended by only trivial inconvenience. But there are some cases of aortic incompetence in which digitalis may be given with advantage. Dr. Barr speaks of cases of "uncomplicated double aortic disease which have been under his treatment for breathlessness interfering with work." It is within my experience that breathlessness is more a marked symptom in simple aortic regurgitation than when that condition is complicated by mitral insufficiency. But it is precisely in these latter cases, when cardiac failure begins to develop, that digitalis is of service. So long as the mitral regurgitation is present so as to allow of a "safety-valve" overflow digitalis may be given with advantage and with safety. In the cases where there is aortic insufficiency alone, the ventricle has to bear the strain of the return pressure; but given mitral incompetence in addition the over-distended ventricle is relieved by an expansile auricle, and accommodating pulmonary veins and plexus. It is said that this relief may be dearly purchased by a shortened life. Possibly so; but remembering the uncertain span of life in all cases of aortic insufficiency, remembering that even in the most promising cases sudden syncope may close the scene, I do not quite see how we are justified in thinking that the comparative comfort afforded by the addition of mitral regurgitation is a condition not to be desired.—*Brit. Med. Jour.*

---

A WARNING ABOUT THE FORCEPS.—In a recent clinical lecture Dr. Goodell said to his class: "Let me warn you, as young men, to resist the temptation of keeping the forceps on too long, in your undue haste or excitement to deliver the woman. Make it your rule to take them off when the head is well down and the perineum begins to bulge, unless the pains have stopped, or the woman is in puerperal convulsions, or she is in any condition demanding prompt delivery. By observing this precept you will at least avoid the accusation that 'the doctor tore her with his instruments'; for indeed it is too true that the physician in his haste to deliver, does often tear his patient either by a too hasty delivery or by pulling parallel with the long axis of the woman's body, instead of following the curve of Carus."—*Practice.*

---

HYSTERIA.—Prof. Da Costa prescribed:

℞. Zinci valerianat........................gr. ij.
Ferri valerianat........................gr. iss.
Ext. belladonnæ........................gr. 1-16.

M., Sig.: One pill three times a day.

The patient should also be given tonics, have a full meat diet, and take exercise in the open air.—*Coll. and Clin. Record.*

TREATMENT OF ECZEMA IN THE FRENCH HOSPITALS.—Dr. Jacquet (*La Semaine Medicale*) advises that attention be paid to the general health, which subject need not be considered here. In eczema in the folds of the skin, or where a parasitic origin is suspected, the writer begins with a calomel salve:

℞. Calomel.............................. grs. viij–xxij.
Oxide of zinc ........................ grs. xlv.
Pure vaseline ........................ ℨiv.

After its application dust on a powder of ten parts of the oxide of zinc and forty parts of talc.

If the disease be obstinate then use the following ointment:

℞. Yellow oxide mercury ............ grs. vij–xv.

Oil of cade ............................ mxv–ʒj.

Pure vaseline ........................ ℨv.

If the eczema is on the hairy scalp then use:

℞. Naphthol, Camphor, Resorcin, } aa ...................... grs. v–xv.

Precipitated sulphur ............... grs. xxx–ℨjss.

Pure vaseline ........................ ℨv.

Nitric acid solution (1:20) may be applied with a camel's-hair brush, or a salve used containing pyrogallic or salicylic acid:

℞. Salicylic acid ........................... grs. vij.
Pyrogallic acid ........................... grs. xv.
Pure vaseline ............................ ʒv.

If these act as irritants one may return to less energetic topical applications. The writer also uses plasters; the best of these contains the oxide of zinc. In rebellious cases one may employ the cod-liver oil as a local application. In pruriginous cases one should use warm solutions of carbolic acid, together with a protective salve of the following consistence:

℞. Ethereal oil of peppermint or carbolic acid .............................. gtts. vij.
Pure vaseline ............................ ʒivss.
Oxide of zinc ............................ ʒiij.

Fissures are touched with a 15 per cent. solution of argentic nitrate.—*Lancet-Clinic.*

TREATMENT OF GONORRHŒAL VAGINITIS.—The woman, if possible, should be kept in bed, and salol and an alkali administered internally for the relief of painful micturition. A lotion of bichloride solution 1:5,000 is used for bathing the outer parts and for injecting into the vagina three or four times a day for the first three days. After this the strength is reduced to 1:10,000. If this injection be painful carbolic acid solution is substituted for it. The injection should be taken with the patient in a recumbent posture. About the third day a speculum can be used and the cervix exposed to view. The parts may then be swabbed with strong carbolic acid or with a solution of bichloride of mercury, and this followed by dusting the parts with powdered iodoform. Later on a five per cent. solution of nitrate of silver may be painted over the cervix and vagina, and the vagina tamponed with iodoform gauze so as to separate its walls, the ends of the tampon being brought down to the vulva. If these are changed every two days the discharge will usually cease within a week or ten days. Before discharging the case as cured, never omit to examine the glands of Bartholini, and if any pus can be made to exude from them you may rest assured that the disease has not been eradicated. They should then be incised under cocaine anesthesia and touched with strong carbolic acid. If there be purulent discharge from the cervix and the Nabothian glands are distended, curette them and apply pure carbolic acid, using the iodoform tampon to prevent reinfection of the vagina.—*The American Journal of Obstetrics.*

---

DISINFECTANT MOUTH WASH.—Dr. Thomas finds the following a pleasant and efficient buccal disinfectant:

R. Thymol..................................gr. iii.
Benzoic acid..............................gr. xl.
Tinct. of eucalyptus....................ʒ iii.
Essence of peppermint..................m x.
Alcohol....................................℥ iii.

M. d.—Sig.: Pour enough into a glass of water to render it turbid, and use as a mouth wash.—*Medical Fortnightly.*

REMOVAL OF NECROTIC AND CARIOUS BONE WITH HYDROCHLORIC ACID.—Dr. Robert T. Morris, of New York, in a paper read before the Virginia State Medical Society, stated that deforming and dangerous operations could be avoided by adopting a method which he thought was now complete. An opening is to be made directly down to the dead bone, and a large sinus formed. All other related sinuses are then made to lead into the large single one if possible. At the end of about a week, when granulation of the walls of the sinus has begun, the cavity is injected with a three-per-cent. solution of hydrochloric acid in distilled water. The frequency of making injections varies with the nature of the case. If the patient is confined to bed, injections can be made every two or three hours. If the patient is going about, injections are made only at night, and the patient is directed to assume a position in bed that will retain the acid solution. This acid injection decalcifies dead bones rapidly, but as only superficial layers are attacked, other experiments have failed because they could not get rid of decalcified bone readily, and the acid solution did not penetrate to deep portions through it. Dr. Morris' plan consists in injecting into the sinus, at intervals of about two days, an acidulated pepsin solution, and this will digest out the decalcified bone in two or three hours. The whole process is then repeated until the sinus closes from the bottom, which it will do when the last dead bone is out.—*Virginia Medical Monthly.*

---

MEDICAL TREATMENT OF PERITYPHLITIS.—The view, which appears to be gradually gaining ground, more especially among surgeons, that once inflammation of the appendix cæci has been diagnosticated these cases should be handed over for surgical treatment, has induced Dr. Saundby, of Birmingham, to put on record fifteen cases of appendicitis which have been under his care during the past six years.

Among these fifteen cases there was only one death—the only case treated surgically—and the post mortem appearances led the writer to believe that life might have been spared had he adhered to purely medical treatment. 86.6 per cent. were cured, and

6.6 per cent. relieved. The average length of the treatment is admittedly long, and a more rapid cure by surgical means might be claimed, though the writer is of opinion that a little time may fairly be sacrificed in view of the inevitable risks of surgical interference. The plan of treatment he adopts is rest, free evacuation of the bowels, hot fomentations or the ice-bag, with the addition, in chronic cases, of repeated blistering over the tumor. He strongly supports the method of treatment by the administration of full doses of sulphate of magnesium which was advocated by a recent American writer, Dr. W. T. Dodge.—*Birmingham Medical Review.*

---

DIAGNOSIS OF INCIPIENT TABES DORSALIS.—Prof. Fournier, of Paris, has made a study of this disease in its incipiency. While it is easily diagnosed when it is well developed, the contrary is quite true in its incipiency. Examination of the gait and the reflexes is quite insufficient, and finer methods must be employed. They are as follows:

1. Westphal's sign, absence of the patellar reflex, is present in about three-fourths of the cases in the pre-atactic stage.

2. Romberg's sign, which, in advanced stages, consists of swaying movements, total loss of the power of equilibrium, and danger of falling when the patient stands with closed eyes, is brought out if one allow the patient to stand, first, a few minutes with closed eyes, and then have him bend either forwards or backwards, to the side, etc. Sometimes one must wait two or three seconds in order to develop this, but the practiced eye will often detect distinct swaying of the trunk of the body. This muscular inco-ordination is deserving of great attention, as it is often the first symptom of disturbed co-ordination.

3. Difficulty in going down stairs is often one of the most regular and sometimes the first sign of ataxia. Patients go down stairs with great care, holding on to the ladder, fearing that they may fall, which would no doubt happen were they without support.

4. Crossing the legs, for example, in sitting, is not done by an atactic in the same manner as a normal person. An atactic throws his leg out over his knee, describing a broad circle. In many patients, the manner in which they seat themselves in an easy chair is significant of tabes.

5. Marching at command may be divided into three parts. If one ask the patient to arise and to set himself immediately in motion, he thinks it over a moment before doing so. There is a short pause between the rising and the first step, or on rising there is a slight swaying, and accessory movements are necessary. If one have him walk off and tell him to halt as soon as he is commanded, he will stop with a slight indecision, a swaying, a slight quaking, often with a slight backward or forward movement of the body, while the well person stops at once. If one ask the patient, while walking, to turn suddenly about, he will manifest a slight indecision, embarrassment, a faulty position, or, in some cases, threatening to fall.

6. Standing on one foot is the most sensitive sign of all, and if one blindfold him he will then exhibit the finest reaction, will sway from side to side, and even threaten to fall over or be obliged to give it up. All of these signs are not infallible, but are of great service in practice in the diagnosis of ataxia.—*Le Bulletin Medical.*

---

THE TREATMENT OF ABDOMINAL WOUNDS.—Michel Wassilieff, discussing the treatment of these wounds (*Revue de Chirurgie*, 1891, No. 11), says: "The position of the surgeon is very difficult in doubful cases. How shall he act when he is ignorant of the condition of the viscera? To make a laparotomy immediately is a serious step, perhaps useless, for the viscera may not be injured. On the other hand, to postpone operation and wait for more serious symptoms is to endanger the life of the patient." He then quotes Augagneur, who claims that the three dangers to life—internal hemorrhage, shock, and peritonitis—do not justify laparotomy; the first and second because they may of themselves cease, and the third because it has no definite symptoms, for those usually ascribed to it may be due to shock. The grounds on which Reclus and Nogues justify operative interference are as follows: Internal hemorrhage; issue of fecal matter and gas from the wound or the distending of the abdomen by gas; and symptoms of peritonitis. They would prefer systematically no operation to always operating immediately, supporting their opinion by statistics. With no operation they find a mortality of 12 per cent. in punctured wounds and 25 per cent. in gunshot wounds, against 24 and 63 per cent., respectively, where there was operative interference. Wassilieff says, however, that the majority of surgeons prefer operating, and that promptness has much to do with success. He cites six cases of his own, four of which recovered without fever after laparotomy, washing with a 1:8000 sublimate solution, and tamponing with iodoform gauze. All were punctured wounds, with injuries of the viscera. The fifth and the sixth cases—both without early dangerous symptoms, on a punctured wound, the other a contusion—died without operation, and section showed peritonitis due to wounds of the viscera. These cases lead him to believe that in all cases where there is a perforating wound—or, as he puts it where a laparotomy has been begun—it should be completed; but he finds it difficult to decide the question in cases of contusion such as the last. Where there exists the least suspicion of a visceral wound he advises immediate operation. He says: "Certainly we would have obtained better results in these last two cases by operating, and therefore my advice is, that it is better to operate without finding visceral lesions rather than commit the fault of neglecting a case that has internal injuries."

He discredits the authority of the statistics quoted, both on the ground of the period that they cover, and also the unwillingness of operators to report unfavorable cases.—*Am. Jour. Medical Sciences.*

---

A FOLDED newspaper placed under the coat in the small of the back is said to be a sure preventive of pneumonia. Now is the time to subscribe. Copies ready for mailing, five cents.—*Siftings.*

NOTES ON 676 CASES OF TYPHOID FEVER.—Mason (*Boston Medical and Surgical Journal*, April 7, 1892) gives interesting statistics of the above number of cases of typhoid fever, treated in the Boston City Hospital in 1890 and 1891. He calls attention to the fallacy of drawing conclusions from a small number of cases. The mortality of the whole number was 10.4 per cent. while two series of 242 cases gave a mortality of only two per cent. It is found that the mortality of 600 cases treated by the same methods differs but little. Eighty per cent. of the cases were admitted between July first and November first. Sixty per cent. were admitted in August, September and October. The mortality of cases under fifteen years was three per cent.; between fifteen and thirty-five, 10.5 per cent. Over thirty-five years it was 18.4 per cent. Delayed admission to the hospital materially increases the mortality; the mortality being highest in those who entered in the fourth week. About twice as many males were admitted as females. The mortality was about two per cent. greater in females than in males. Intestinal accidents occurred in six per cent. of the cases. He compares this series with a series of 1,173 cases treated by Brand's method, and finds his intestinal accidents less than the series treated by cold baths. He believes their higher general death rate 10.4 per cent. compared with 7.84 per cent. to be due to the fact that Brand's method prevents pyrexial exhaustion, which is so fatal in females. The therapeuties of the 676 cases was cold sponging, use of phenacetine in high temperature, laxatives, preferably salines in the first week, the use of naphthol, salol and other internal antiseptics. Stimulants, he believes, should not be used too early, nor to too great an extent. He believes that careful attention to the digestion and to the eliminating organs, a free supply of drinking water, correct estimation of danger signals, and prompt application of such measures as each case may demand, are of more importance in the management of this malady than any systematic method. But the mortality from typhoid has decreased the world over within the last twenty-five years, and Brand's system of bathing is thought by the best observers to be an important factor in this reduction.—*Dietetic Gazette.*

THE METROPOLITAN, AND THE STATE JOURNAL.—"The reader who sits down to a cosmopolitan medical journal with its overloaded pages, beautifully printed, prompt in its issues, enjoys this privilege and luxury because of the very large subscription list and advertising patronage, only possible to the journals published in the densely populated regions of the North and West. These worthy contemporaries have their place in the medical world, and they perform their mission with a zeal and with such merit as not only to add reputation to the editors and publishers, but to American medicine. As broad as their field is, they have never reached the masses of the profession of the more sparsely settled regions in the South, nor have they been able to propagate the medical reform needed in different States in a way that would shape legislation or give form to public opinion. *Per contra* it has been very remarkable that in three States where medical journals of the highest literary-scientfic order are published—Pennsylvania, Massachusetts and New York—the State laws regulating medical education are of the most primitive and ineffectual kind—in Massachusettes as good as no law. Illinois, with its goodly array of medical journals, relegated the conflict for medical education to its State Board of Health under the admirable leadership of Rauch. So that whatever has been the province of the metropolitan journals, they have never been able to do what a State medical journal could do with a united profession at its back, in solving the problems incident to a higher development of education. Another remarkable thing to notice, that at the very centers of medical education in the cities where our young men thronged to be taught, little could be done in determining a standard of qualifications which all recognize as being just and proper.—*N. C. Medical Journal.*

---

THE OPERATIVE TREATMENT OF THE ENLARGED PROSTATE.—Keyes (*Medical Record*, vol. xl., No. 18) arrives at the following conclusions in regard to the treatment by operation of prostatic hypertrophy :

1. Prostatectomy is justifiable, and does what nothing else can. 2. The perineal operation is somewhat less severe, but decididly less reliable than the supra-pubic ; it should rarely be preferred, unless there be urethral complications. In very feeble men it may still be elected. 3. The operation is not justifiable, with present statistics, if the patient can be comfortable in catheter life. 4. No physical conditions of the parts or of the patient short of a practically moribund state contra-indicates operation. By it in desperate cases life is often actually saved, although the operation is a grave one and its mortality high. 5. With the rongeur—better than any instrument—the bladder outlet can be lowered, and polypoid or interstitial growths jutting into the prostatic sinus can be removed, and these points are more essential to a successful operation than in the taking away of a large portion of the prostatic bulk. The instrument next in value is the curved scissors, but the skilled finger is most important of all. Most of the work has to be done by the aid of touch, as the bleeding soon becomes free and renders visual inspection impossible. 6. Diuretin, perhaps, is of value when the kidneys are damaged. It certainly does no harm. 7. Chloroform alone should be used as an anæsthetic, for the sake of the kidneys.—*Am. Jour. Med. Sciences.*

---

CHRONIC ENDOMETRITIS.—* * * In chronic endometritis, constitutional treatment is indicated, a tonic for instance like the following:

℞. Ferri sulph............................grs. xvi.

Acid sulph. dil.......................ʒ i.

Magnes. sulph.......................℥ ii.

Aquæ ...................................O i.

M. Sig.—Tablespoonful in water, on an empty stomach.

Or, ℞. Sodi et pot. tart.....................℥ ii.

Vini ferri amaræ.........................℥ ii.

Acid tartaric..............................ʒ iii.

Aquæ.......................................℥ xiv.

M. Sig—Two tablespoonfuls before each meal.

—*Englemann.—Med. Fortnightly.*

PEROXIDE OF HYDROGEN IN SURGERY.—1. Hydrogen peroxide is a positive germicide and a possible stimulant to granulating tissues.

2. Owing to its especial property of eliminating oxygen, it is of unparalleled value in the distention of suppurating sinuses and cavities, especially in the mastoid region, or where it is almost impossible to reach unhealthy surfaces by other means.

3. The diluted solution is perfectly harmless and can with safety be used in any quantity.

4. The strong concentrated solution, syrupy in consistence, is a direct irritant to all tissues, and should never be used.

5. It possesses healing and cleansing qualities as well as those germicidal in nature.

6. When exposed to light it loses strength ; care should therefore be exercised in keeping the bottles well stoppered with rubber corks, and in a cool, dry place.

7. Fibrin, cellular tissues and some metals instantly decompose it. In contact with sugar and starch it eliminates carbon dioxide ($CO_2$).

8. In washing suppurating surface, it should be used until oxidation ceases, thus showing a complete destruction of all existing purulent material.—*Dr. Engleton in* "*Notes on New Remedies.*"

---

CANNABIS INDICA.—Cannabis indica has proven very useful in the treatment of melancholia and mania. It is of great value in the treatment of chorea when arsenic fails. It may be combined with chloral with advantage in such cases. In migraine the drug is of great value ; a pill containing a quarter of a grain of the extract, with or without the same amount of phosphide of zinc, will often check an attack immediately, and if the pill is given twice a day, continuously, the severity and frequency of the attacks are often much diminished. Patients who have been incapacitated for work from the frequency of the attacks have been enabled by the use of cannabis indica to resume their employment. The drug is also a valuable gastric sedative in cases of ulcer of the stomach and gastrodynia. It may be combined with nitrâte of silver, and it increases the efficacy of the latter. It is also a valuable hypnotic.—*Br. Med. Jour.*

MORTALITY FROM CARCINOMA.—The mere fact that carcinoma causes more deaths in the United States in one year than the sum-total of deaths due to erysipelas, tetanus, hydrophobia, lightning, typhlitis, gunshot wounds, joint disease, together with other well-known surgical affections, conveys at once an idea of the wide dimensions of our subject. When we learn that carcinoma causes nearly half as many deaths in one year in the United States as are caused by accidents and injuries of all kinds and descriptions, the importance of our subject is imperatively forced upon us.

For example, in the United States, in one year, there were over thirteen thousand deaths from carcinoma, of which there were twice as many deaths among females as among males. There are over fourteen thousand people in the United States dying each year from carcinoma. There were one thousand three hundred and eighty-seven cases of death from carcinoma of the breast alone in this country during the year 1880.—*Dr. F. S. Dennis.*

---

SYPHILIS IN PHYSICIANS.—Dr. James D. White says that he has notes of fifteen cases of syphilis in physicians who were inoculated upon their hands while engaged in professional duties (*Boston Med. and Surg. Jour.*, in *Med. and Surg. Rep.*, April 23). Three of these died of the disease. All of them suffered greatly in mind, and were more or less physically disabled or disfigured for a time. He suggests: First, always wash one's hands immediately after an interview with a patient who has, or is suspected to have, syphilis, without regard to the condition of the parts touched, or even if not handled at all. This should be made an invariable custom. Second, the physician should regard every persistent sore upon himself, of whatever seat and however innocent apparently, as a possible lesion of tuberculosis or syphilis, and seek competent advice without delay. Such advice is never found at home.—*College and Clinical Record.*

TREATMENT OF GALL-STONE COLIC BY GLYCERINE.—Dr. Ferrand has presented a work on this subject to the French Academy of Medicine. His conclusions are as follows:

1. Glycerine, administered by the stomach, is absorbed unchanged by the lymphatics, especially by the vessels which run from the stomach to the hilus of the kidney and the gall-bladder. It is found even in the blood of the subhepatic veins.

2. It is a powerful cholagogue and a precious remedy in the treatment of gall-stone colic.

3. A relatively large dose (twenty to thirty grammes, one-half to one ounce) will bring the attack to an end.

4. A small dose (five to fifteen grammes, one and a quarter to four drachms), taken in a little alkaline water, will act as a prophylactic.

5. Glycerine, without being a lithotriptic, is the remedy, *par excellence*, in gall-stone colic.—*La Semaine Medicale.*

---

DO ORGANIC DISEASES OF THE HEART PRECLUDE THE USE OF CHLOROFORM IN PARTURITION?—In the *Med. and Surg. Reporter*, Dr. L. Ridgeway Barber discusses ably the question of administering chloroform in labor to women who have organic heart disease. He concludes that chloroform by inhalation can and will, if properly administered, save the lives of parturient females, suffering from organic disease, when death seems imminent from over-stimulation of its ganglia through reflex nervous action. Organic heart disease, then, does not preclude the use of chloroform in labor, but rather is a condition calling for its careful administration.—*West. Med. Rep.*

---

CHOREA WITH VALVULAR HEART DISEASE.—Prof. J. M. DaCosta gave this prescription with excellent results:

℞ Tinct. belladonnæ ...................... ʒ i

Liq. potassii arsenitis .................. ʒ ii

Syr. hypophosphitis comp. q. s. ad ...... ℥ iii

M. d.—Sig.: A teaspoonful to be taken three times daily.

—*Medical News.*

THE DIAGNOSIS AND TREATMENT OF HIP JOINT DISEASE.—Six cases are reported in detail and the following conclusions are drawn :

1. An early diagnosis can be made by any one who examines the case carefully, and who familiarizes himself with the functions of a sound joint.

2. The necessity of regarding a case as chronic and therefore requiring prolonged protection of the joint.

3. The comfort that any patient may derive from an apparatus that is made to fit.

4. The benign progress of a case thus protected.

5. The importance of maintaining parallelism and equality of the limbs at all times, and under all circumstances.

6. The advantages of out-of-door life, which cannot be secured by bed treatment.

7. The necessity of excision of the hip when well-directed efforts at securing rest and protection to the joint have failed.—*V. P. Gibney, in Boston Medical and Surgical Journal.*

---

FOR ASTHMA.—Dr. Holland in the *Times and Register* recommends :

℞. Acid hydrocyanic dil........................ *m* xv.

Tinct. lobeliæ.............................. ʒiij.

Syr. scillæ comp.......................... ℥ss.

Spt. lavand. comp ........................ ʒiij.

Syr. simp ...................................... ℥j.

M.—Sig. One teaspoonful to be taken when attacks come on, and repeated within an hour if not relieved.

---

IT is said that the Jews have a less mortality, fewer still-born less illegitimacy, less crime, less insanity, and greater longevity than the Christians.

